# Exosymbiotic microbes within fermented pollen provisions are as important for the development of solitary bees as the pollen itself

**DOI:** 10.1002/ece3.8788

**Published:** 2022-04-06

**Authors:** Prarthana S. Dharampal, Bryan N. Danforth, Shawn A. Steffan

**Affiliations:** ^1^ Department of Entomology University of Wisconsin Madison Wisconsin USA; ^2^ Department of Entomology Cornell University Ithaca New York USA; ^3^ USDA‐ARS Vegetable Crops Research Unit Madison Wisconsin USA

**Keywords:** bee–microbe symbioses, exosymbionts, larval fitness, oligolege, pollen provisions, polylege, solitary bees

## Abstract

1. Developing bees derive significant benefits from the microbes present within their guts and fermenting pollen provisions. External microbial symbionts (exosymbionts) associated with larval diets may be particularly important for solitary bees that suffer reduced fitness when denied microbe‐colonized pollen.
2. To investigate whether this phenomenon is generalizable across foraging strategy, we examined the effects of exosymbiont presence/absence across two solitary bee species, a pollen specialist and generalist. Larvae from each species were reared on either microbe‐rich natural or microbe‐deficient sterilized pollen provisions allocated by a female forager belonging to their own species (conspecific‐sourced pollen) or that of another species (heterospecific‐sourced pollen). Our results reveal that the presence of pollen‐associated microbes was critical for the survival of both the generalist and specialist larvae, regardless of whether the pollen was sourced from a conspecific or heterospecific forager.
3. Given the positive effects of exosymbiotic microbes for larval fitness, we then examined if the magnitude of this benefit varied based on whether the microbes were provisioned by a conspecific forager (the mother bee) or a heterospecific forager. In this second study, generalist larvae were reared only on microbe‐rich pollen provisions, but importantly, the sources (conspecific versus heterospecific) of the microbes and pollen were experimentally manipulated.
4. Bee fitness metrics indicated that microbial and pollen sourcing both had significant impacts on larval performance, and the effect sizes of each were similar. Moreover, the effects of conspecific‐sourced microbes and conspecific‐sourced pollen were strongly positive, while that of heterospecific‐sourced microbes and heterospecific‐sourced pollen, strongly negative.
5. Our findings imply that not only is the presence of exosymbionts critical for both specialist and generalist solitary bees, but more notably, that the composition of the specific microbial community within larval pollen provisions may be as critical for bee development as the composition of the pollen itself.

## Graphical abstract

We report that exosymbiotic microbes within larval pollen provisions are critical for the development of solitary bees, regardless of their foraging strategy. Furthermore, the particular microbial community composition within pollen provisions is as critical as the pollen source itself, both factors being significant and co‐equal drivers of bee fitness.

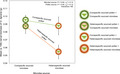

## 1 INTRODUCTION

Solitary bees, which represent the vast majority of global bee diversity (Danforth et al., 2019), are among the most important insect pollinators within agricultural and seminatural landscapes (Garibaldi et al., 2013; Klein et al., 2018; Williams & Kremen, 2007). Over the past decade, solitary bee populations have been facing rapid declines (Powney et al., 2019) due to increasing threats from pesticide overuse (Azpiazu et al., 2019), novel diseases (Ravoet et al., 2014), and landscape fragmentation (Kline & Joshi, 2020). Aspects of life history such as pollen specialization (Biesmeijer et al., 2006; Bommarco et al., 2010) and limited foraging range (Greenleaf et al., 2007) render this species‐rich group of wild pollinators more susceptible to such risk factors (Burkle et al., 2013; Kremen & Ricketts, 2000; Sgolastra et al., 2019). Along with these well‐known stressors, growing evidence suggests that the partnership between solitary bees and their microbial symbionts may play an important role in determining bee fitness. Collectively known as the solitary bee microbiome (Voulgari‐Kokota et al., 2019), symbiotic microbes have been shown to perform significant nutritive (Dharampal et al., 2019, 2020; Gilliam et al., 1984; Steffan et al., 2019) and protective functions for the developing larvae (Keller et al., 2018; McFrederick et al., 2012), shaping the overall fitness of these critical pollinators.

While several studies have reported that the microbiome within the guts of adult social bees plays a significant role in maintaining bee fitness (Kwong et al., 2017; Kwong & Moran, 2016; Raymann & Moran, 2018), others suggest that the function of the gut microbiome alone is not sufficiently predictive of brood outcome (Gilliam et al., 1990; Martinson et al., 2012). In fact, mounting evidence across diverse bee species suggests that microbes occurring outside the bee gut, especially those within pollen/nectar provisions, harbor bacteria and fungi (Gilliam, 1997; McFrederick et al., 2013; Pimentel et al., 2005; Rosa et al., 2003; Yoder et al., 2017) that may be vital to larval nutrition (Steffan et al., 2019; Vannette et al., 2012), immune function (Kaltenpoth & Engl, 2014; McFrederick et al., 2014), and overall fitness (Cohen et al., 2020; Dharampal et al., 2019; Dharampal, Diaz‐Garcia, et al., 2020; Rothman et al., 2020; Steffan et al., 2017; Voulgari‐Kokota, McFrederick, et al., 2019; Voulgari‐Kokota et al., 2020). This phenomenon appears to be broadly applicable to global bee fauna, whether social (Anderson et al., 2014; Gilliam et al., 1989) or solitary (Gilliam et al., 1984; Graystock et al., 2017; McFrederick & Rehan, 2016).

The microbiome of larval pollen provisions may be especially critical for the maturation of solitary bees since they have limited opportunities of acquiring microbial symbionts through brood care and/or social interactions with other nestmates (Keller et al., 2020; Voulgari‐Kokota et al., 2019). Primarily sourced from the environment (McFrederick et al., 2012, 2016; Rothman et al., 2019; Voulgari‐Kokota et al., 2018), some of these external symbionts (exosymbionts) are thought to be involved with the fermentation and/or preservation of pollen–nectar provisions prior to larval consumption (Gilliam et al., 1984; Lozo et al., 2015; McFrederick et al., 2018; Steffan et al., 2019). Empirical data from diet reconstruction studies suggest that the heterotrophic microbes enmeshed within the pollen provisions literally consume and assimilate the resources within plant biomass (i.e., pollen), effectively displacing plant biomass with that of their own (Steffan & Dharampal, 2019; Steffan et al., 2017). Because the microbial communities are able to access and consolidate pollen nutrients (amino acids, lipids, and non‐structural carbohydrates), these microbes likely serve as conduits for nutrient transfer from pollen to larval biomass, directly influencing the brood outcome among solitary bees (Dharampal, Hetherington, et al., 2020; Steffan & Dharampal, 2019). In fact, tracing microbial ‘fingerprints’ using trophic biomarkers suggests that these exosymbionts may represent a direct and dominant source of proteins and lipids for the developing bees, their contribution often exceeding that of pollen itself (Dharampal et al., 2019; Steffan et al., 2019).

An individual solitary bee nest, which is provisioned by a single foraging female, contains several discrete brood chambers, each stocked with a one‐time supply of pollen, nectar, and sometimes floral oils (Danforth et al., 2019). The brood chambers host a diverse community of biologically important microbes, including specialized taxa that are reportedly involved with pollen degradation, digestion, and preservation (Cohen et al., 2020; Pimentel et al., 2005; Voulgari‐Kokota et al., 2020). While bees that progressively provision their larvae with nutritional resources (e.g., honey and bumble bees) can store the pollen–nectar blend for hours to days prior to larval consumption (Anderson et al., 2014), the nest‐stored pollen of massprovisioning solitary bees can undergo fermentation for several weeks (Gilliam et al., 1984). The extended storage duration likely provides opportunities for microbes to proliferate to high abundances (Batra et al., 1973; Miliczky, 1985; Roberts, 1971), and enzymatically transform or at least strongly influence the nutritive quality of the provision itself. For larval solitary bees, there appears to be an increased dependence on the resident microbes within the fermenting pollen provision (Voulgari‐Kokota, McFrederick, et al., 2019), possibly since pollen provisions in such mass provisioning species tend to ferment the pollen for a much longer period than progressive provisioners (Danforth et al., 2019).

Based on their foraging strategy, some species of solitary bees are characterized as pollen specialists (oligoleges), foraging on a few related plant species belonging to the same family, whereas others as pollen generalists (polyleges) that have a broader host plant range (Cane & Sipes, 2006). Many plant species are known to host both oligoleges and polyleges, and the pollen collected by oligolectic and polylectic bees can span a spectrum of nutritional quality as determined by its protein content (Roulston & Cane, 2000; Roulston et al., 2000). However, past research indicates that pollen collected by oligoleges is often of lower quality and/or may contain toxic compounds (Dharampal, Hetherington, et al., 2020; Weiner et al., 2010), making it unfit as the sole source of food for polylectic larvae (Brochu et al., 2020; Cane, 2018; Spear et al., 2016; Vanderplanck et al., 2020). The ability of oligolectic larvae to utilize such low‐quality pollen has been attributed to their digestive physiology (Dobson & Peng, 1997; Praz et al., 2008). However, a previous study utilizing *Osmia ribifloris*, an Ericaceae specialist, has shown that larvae appear more dependent on the function of the natural microbiota (conspecific microbes) associated with the maternally allocated provisions (conspecific pollen), much more so than the identity of the host plant pollen itself (Dharampal, Hetherington, et al., 2020). This finding suggests that the conspecific microbes embedded within the conspecific pollen, rather than the identity of the pollen per se, play an important role in larval nutrition among oligoleges (Dharampal, Hetherington, et al., 2020; Weiner et al., 2010).

A vast body of literature has documented the presence of diverse exosymbiotic microbes associated with solitary bee species (Christensen et al., 2021; Dew et al., 2020; Gilliam, 1997; Graystock et al., 2017; Keller et al., 2013; McFrederick & Rehan, 2016; Rothman et al., 2020). It has been speculated that these exosymbionts likely perform vital nutritive and defensive functions that strongly influence bee health. For instance, the natural conspecific microbiota within the pollen provisions of oligolectic bees may play a critical role in larval development by enhancing the nutritive value of the low‐quality pollen collected by oligolectic foragers (Dharampal, Hetherington, et al., 2020). While the microbiome of solitary bees has received growing attention, the nature and magnitude of the fitness benefit provided by the external microbial symbionts has seldom been empirically quantified and compared across bee foraging strategies.

**FIGURE 1:**
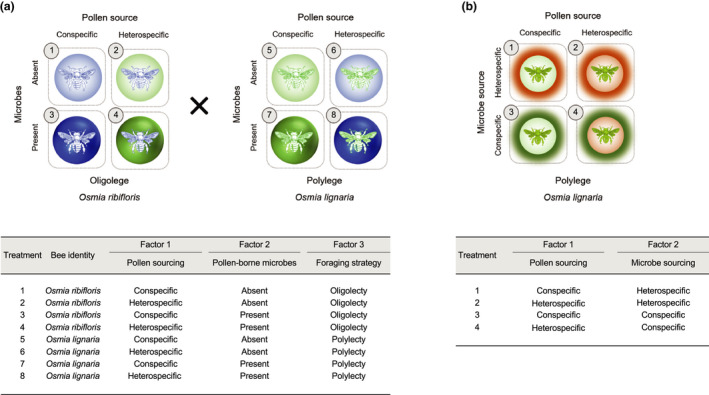
(a) Schematic representation of the factorial experimental design for Study 1. Blue and green silhouetted bees represent the oligolege, *Osmia ribifloris*, and the polylege, *Osmia lignaria*, respectively. Dark and light blue circles represent natural and sterilized pollen provisions of *Osmia ribifloris*, respectively. Dark and light green circles represent natural and sterilized pollen provisions of *O*. *lignaria*, respectively. (b) Schematic representation of the factorial experimental design for Study 2. Green bee represents the polylege, *O*. *lignaria*. The color of the circle indicates the source of pollen; light green and light orange circles represent sterilized *O*. *lignaria* (conspecific‐sourced) and *O*. *cornifrons* (heterospecific‐sourced) pollen, respectively. The color of the circle outlines indicates the source of microbes; green and orange outlines represent microbes associated with *O*. *lignaria* (conspecific‐sourced), and *O*. *cornifrons* (heterospecific‐sourced), pollen provisions respectively

In this study, we hypothesized that the presence of conspecific‐sourced microbes would have a greater positive impact on larval fitness than conspecific‐sourced pollen, and that the magnitude of this beneficial effect would be larger among oligoleges than polyleges. To test this hypothesis, we conducted two separate experiments:

In the first study (Study 1), we reared larvae of the oligolege, *Osmia ribifloris*, and the polylege, *Osmia lignaria*, on their own conspecific‐sourced pollen (i.e., *O*. *lignaria* on *O*. *lignaria* pollen, and *O*. *ribifloris* on *O*. *ribifloris* pollen) and heterospecific‐sourced pollen (i.e., *O*. *ribifloris* on *O*. *lignaria* pollen, and *O*. *lignaria* on *O*. *ribifloris* pollen), in the presence and absence of their respective pollen‐associated microbiota (Figure 1a). We predicted that larvae would perform best when they had access to both conspecific‐sourced pollen and conspecific‐sourced microbes, with the effect size of microbes being greater than that of pollen. We also predicted that the beneficial effect of microbe availability would be greater for the oligolege than the polylege.

In a follow‐up study (Study 2), we manipulated both the source of pollen (conspecific‐sourced pollen versus heterospecific‐sourced pollen) and the source of microbes (conspecific‐sourced microbes versus heterospecific‐sourced microbes) used to reinoculate the pollen. Unlike in Study 1, which tested *the effect of microbe presence*/*absence* within pollen provisions, Study 2 examined *the effect of microbial sourcing*. To these ends, Study 2 included microbes in every pollen provision and explicitly tested whether it mattered for bee larvae if their diets contained microbes from conspecific or heterospecific sources. Simultaneously, the study also tested the effect of pollen sourcing; thus, Study 2 allowed for an examination of the main and interactive effects of microbial and pollen sourcing on bee fitness. For this experiment, *O*. *lignaria* larvae were reared only on microbe‐rich pollen diets, the pollen, and/or microbes being obtained either from a conspecific *(O*. *lignaria)* or a heterospecific source. Heterospecific‐sourced pollen and heterospecific‐sourced microbes were obtained from another polylectic congener, *Osmia cornifrons*, which was abundant during the time when the study was conducted. In simultaneously examining the relative importance of pollen and microbe sources for larval performance of *O*. *lignaria*, we predicted that larval fitness would be highest when both pollen and microbes were sourced from a conspecific forager, and lowest when sourced from a heterospecific (Figure 1b).

## 2 METHODS

### 2.1 Bees and pollen provisions

Wild‐collected bees from Washington, Utah, and New York were used for the studies described here. Nesting reeds of *O*. *ribifloris* and *O*. *lignaria* were received in overnight shipments from commercial suppliers in April–June 2018 (NativeBees.com and Crown Bees, respectively). *Osmia ribifloris* were collected in Kaysville, Utah, where the nesting females forage almost entirely on *Mahonia aquifolium* (Oregon grape) found within the region. *O*.* lignaria*, which typically forage on pollen and nectar from a wide array of orchard plants (Bosch & Kemp, 2000), were collected around Woodenville, Washington. Nesting reeds of *O*. *cornifrons* were collected from a natural nesting site near Ithaca, NY, by BND. Once in the laboratory, the nesting reeds were dissected using aseptic technique and the eggs of *O*. *ribifloris* and *O*. *lignaria* were sexed based on previously published guidelines (Bosch, 1994). Since *Osmia* sp. provision larger pollen masses for females than males, and since males are much more abundant, we chose to only use the provisions from male cells for this study (Table S1 and S2).

Male pollen provisions and eggs of *O*. *ribifloris* and *O*. *lignaria* were individually weighed and the pollen/nectar provisions from the same species were pooled into a single mass in order to eliminate variation among individuals in pollen, nectar, or microbial content. From this pooled mass, half was sterilized to obtain sterile host pollen, while the remaining half was left untreated. Similarly, pollen provisions collected from all nesting reeds of *O*. *cornifrons* were pooled and divided into sterile and natural fractions. Pollen was sterilized using previously detailed methods using a 95% ethanol soak and overnight drying under germicidal UV light after lyophilization. Past studies have empirically verified the nutritional integrity of sterilized pollen using this technique (Dharampal et al., 2019; Dharampal, Diaz‐Garcia, et al., 2020; Steffan et al., 2017). Dry sterilized pollen was rehydrated using sterile water based on the natural moisture content of maternally allocated provisions of each species (~20% for *O*.* ribifloris*, ~13.5% for *O*. *lignaria*, and ~6% for *O*.* cornifrons)*.

### 2.2 Experimental design

*Study 1*: The experiment was conducted using the oligolege, *O. ribifloris*, and the polylege *O. lignaria* and consisted of eight diet treatments based on a fully crossed 2 × 2 × 2 factorial design (*N* = 10 larvae/treatment). Each factor consisted of two levels; Factor 1: Pollen source (levels: Conspecific; Heterospecific), Factor 2: Pollen‐borne microbes (levels: Present; Absent), and Factor 3: Foraging strategy (levels: Oligolectic; Polylectic). Based on this design, only four of the eight unique diet treatments also contained the associated microbes (Treatment 3, 4, 7, and 8), while the others did not (Treatments 1, 2, 5, and 6) (Figure 1a; Table S1).

*Study 2*: The experiment was conducted using the polylege, *O. lignaria*, and consisted of four diet treatments based on a fully crossed 2 × 2 factorial design (*N* = 10 larvae/treatment). Each factor consisted of two levels; Factor 1: Pollen source (levels: Conspecific; Heterospecific), Factor 2: Microbe source (levels: Conspecific; Heterospecific). Based on this design, all four treatments consisted of pollen provisioned either by a conspecific (*O. lignaria*) or heterospecific (*O. cornifrons*) forager that was sterilized and subsequently reinoculated with microbial populations associated with either of the two pollen sources prior to larval consumption. Therefore, unlike in Study 1, larval diets for all treatments in Study 2 contained pollen‐associated microbes. However, the source of microbes, whether conspecific or heterospecific, was experimentally manipulated.

To prepare the diet treatments for Study 2, we combined 80% (w/w) sterilized pollen sourced from either the conspecific (*O. lignaria*) or heterospecific (*O. cornifrons*) forager with 20% (w/w) natural pollen provisions from *O. lignaria* and *O. cornifrons* as an inoculum containing conspecific and heterospecific microbes, respectively. Thus, each sterilized pollen provision, whether sourced from a conspecific or heterospecific forager, was colonized with either conspecific‐sourced or heterospecific‐sourced microbes and as the study progressed, the microbial community in a given pollen provision could propagate throughout the provision. Inoculating sterilized *O. lignaria* pollen with *O. lignaria* microbes represented a diet containing both the ‘right pollen’ and the ‘right microbes’ (i.e., conspecific‐sourced pollen and conspecific‐sourced microbes), whereas *O. lignaria* pollen‐inoculated *O. cornifrons* microbes simulated a diet with the ‘right pollen’ but ‘wrong microbes’ (i.e., conspecific‐sourced pollen and heterospecific‐sourced microbes). Similarly, sterilized *O. cornifrons* pollen inoculated with *O. cornifrons* microbes represented a diet of ‘wrong pollen’ and ‘wrong microbes’ (i.e., heterospecific‐sourced pollen and heterospecific‐sourced microbes), whereas *O. cornifrons* pollen inoculated with *O. lignaria* pollen represented the ‘wrong pollen’ but ‘right microbes’ (i.e., heterospecific‐sourced pollen and conspecific‐sourced microbes). While the diet treatment with the ‘right pollen’ and the ‘right microbes’ (*O. lignaria* pollen with *O. lignaria* microbes) most closely mimicked the natural diet for *O. lignaria* larvae, the ‘wrong pollen’ and ‘wrong microbes’ (*O. cornifrons* pollen with *O. cornifrons* microbes) was the most contrived (Figure 1b; Table S2).

For both studies, larvae were reared from egg to prepupal stage within sterile 48‐well plates based on previously described methods (Dharampal et al., 2018). Separate plates were used for each treatment to minimize the risk of cross‐contamination. The weights of the rehydrated sterilized pollen and natural pollen fractions were adjusted such that when combined, the end weight of the reconstituted pollen provision was approximately equal to that of a naturally allocated provision (fresh weight ~0.35 g and ~0.37 g for *O. ribifloris* and *O. lignaria*, respectively) (Table S1 and S2). All procedures were carried out inside a biosafety cabinet using standard aseptic technique. The plates were loosely taped and maintained under dark conditions at 22°C in an incubator. Larvae were observed daily until they reached the prepupal stage, characterized by the completion of a pale silken cocoon. To minimize handling stress and reduce contamination risk, all surviving larvae were aseptically weighed on days 1, 10, 15, and 20 by placing them on pre‐sterilized aluminum weigh boats using a standard laboratory microbalance located inside a biosafety cabinet. Larval fitness components were assessed using survivorship and biomass.

### 2.3 Statistical analyses

*Study 1*: Separate two‐way ANOVAs were conducted to test the impact of main and interactive effects of the independent variables, pollen source (two levels: conspecific; heterospecific) and foraging strategy (two levels: oligolectic; polylectic), on the dependent variables, prepupal biomass and developmental time of larvae reared on natural pollen. Median survival time and distribution was compared across all treatments using the log rank and Gehan–Breslow–Wilcoxon tests. Proportional hazard rate based on time to death for each bee species was modeled using Cox regression analysis (Katz & Hauck, 1993). The end point was set at 25 days, and the covariates in the model included pollen‐borne microbes (0 = present; 1 = absent) and source of pollen (0 = conspecific; 1 = heterospecific).

*Study 2*: Differences in the rate of biomass accrual for *O. lignaria* larvae were analyzed using a repeated measures ANOVA for the first three time steps, on days 0, 5, and 10. Since there were fewer than two surviving larvae in one or more of the treatments beyond day 10, and no further statistical tests could be conducted reliably beyond this time point. A two‐way ANOVA was conducted to test the impact of main and interactive effects of the independent variables, pollen source (two levels: conspecific; heterospecific) and microbial source (two levels: conspecific; heterospecific) on the dependent variable, larval biomass. Hedge's g estimate of effect size was calculated to ascertain the impact of microbe sourcing across conspecific‐sourced and heterospecific‐sourced pollen types, and that of pollen sourcing across conspecific‐sourced and heterospecific‐sourced microbes for larval biomass. The confidence level for statistical significance was set at 95%, *p* = .05. All statistical analyses were conducted using SPSS v26 (IBM).

## 3 RESULTS

*Study 1*: Larvae of both *O. ribifloris* and *O. lignaria* suffered high mortality when reared on pollen provisions without microbes as indicated by Kaplan–Meier survivorship analysis. For *O. ribifloris*, there were no survivors among larvae reared on sterilized heterospecific‐sourced pollen, while only two larvae survived when reared on sterilized conspecific‐sourced pollen. There were no survivors among *O. lignaria* when reared on sterilized pollen from either conspecific or heterospecific sources. Such qualitative data showing dramatic mortality among larvae reared on sterilized pollen suggested that microbes were essential for larval development for both bee species across both diets. The insufficient sample size for surviving larvae from the sterilized diet treatments prevented us from conducting further statistical analyses to quantify the impact of removing pollen‐associated microbes on larval biomass across all eight treatments.

Results from the two‐way ANOVA indicated no statistically significant interaction or main effects of pollen source and foraging strategy on prepupal biomass of larvae reared on natural pollen. The main effect of pollen source was statistically non‐significant (*F*_1,32_ = 0.052, *p* = .821, η_p_^2^ = 0.002) with larvae consuming natural conspecific‐sourced and natural heterospecific‐sourced pollen weighing 0.141 ± 0.019 g, (mean ± 1 SD) (*N* = 18) and 0.139 ± 0.027 g (*N* = 18), respectively. The main effect of foraging strategy was statistically non‐significant (*F*_1,32_ = 0.952, *p* = .337, η_p_^2^ = 0.029) with oligolectic and polylectic larvae weighing 0.136 ± 0.016 g (*N* = 19) and 0.144 ± 0.030 g (*N* = 17), respectively. The interaction term was also statistically non‐significant (*F*_1,32_ = 0.414, *p* = .524, η_p_^2^ = 0.013). Similar results were noted for developmental time for larvae reared on natural pollen. The main effect of pollen source was statistically non‐significant (*F*_1,32_ = 0.053, *p* = .471, η_p_^2^ = 0.016) with larvae consuming natural conspecific‐sourced and natural heterospecific‐sourced pollen taking 17.00 ± 1.79 d (*N* = 18) and 17.44 ± 2.15 d (*N* = 18) to complete development, respectively. The main effect of foraging strategy was statistically non‐significant (*F*_1,32_ = 2.10, *p* = .157, η_p_^2^ = 0.061), with oligolectic and polylectic larvae taking 16.79 ± 2.02 d (*N* = 19) and 17.71 ± 1.79 d (*N* = 17) to complete development, respectively. The interaction term was also statistically non‐significant (*F*_1,32_ = 0.363, *p* = .551, η_p_^2^ = 0.011).

**FIGURE 2:**
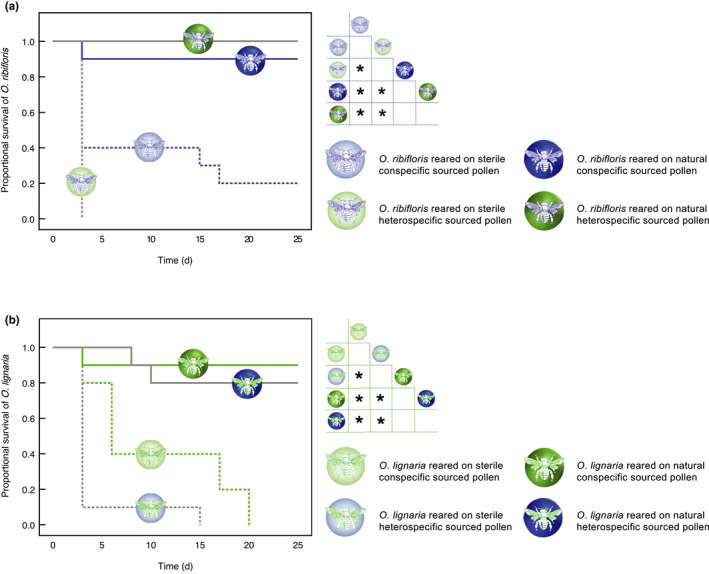
Kaplan–Meier survival plot of (a) *Osmia ribifloris* and (b) *Osmia lignaria* across diet treatments. Inset symbols along each survival curve correspond to individual treatments for each bee species. Survival analysis indicates significant differences in the median survival time across all eight treatments (log rank test: χ^2^_(7)_ = 75.87, *p* < .0001, Breslow–Wilcoxon test: χ^2^_(7)_ = 64.89, *p* < .0001). Survival distribution indicates significant differences across diet treatments within each species; for (a) *Osmia ribifloris*, Gehan statistic: *p* < .001; log rank test: *p* < .001; and for (b) *Osmia lignaria*, Gehan statistic: *p* < .001; log rank test: *p* < .001. Inset grids next to each survival plot indicate pairwise comparisons of survival distribution (*p < .05)

The end date for survival analysis was set at day 25, by which point all surviving larvae had completed larval development. Survival analysis indicated significant differences in the median survival time across all eight treatments (log rank test: χ^2^_(7)_ = 75.87, *p* < .0001, Gehan–Breslow–Wilcoxon test: χ^2^_(7)_ = 64.89, *p* < .0001). Survival distribution also showed significant differences in median survival time based on treatment within each species (Gehan statistic: *O. ribifloris*: *p* < .001; *O. lignaria*: *p* < .001, log rank test: *O. ribifloris*: *p* < .001; *O. lignaria*: *p* < .001). Pairwise comparisons for *O. ribifloris* indicated that across all four treatments, survival time was significantly lowest for larvae reared on heterospecific‐sourced pollen without microbes (sterilized *O. lignaria* pollen). However, survival time was comparable for larvae reared on conspecific‐sourced (natural *O. ribifloris* pollen) and heterospecific‐sourced (natural *O. lignaria* pollen) pollen that contained the respective microbiota (Figure 2a; Table S3). Similar trends were noted for *O. lignaria* as well, wherein survival time was significantly lowest for larvae reared on heterospecific‐sourced pollen without microbes (sterilized *O. ribifloris* pollen), and was comparable for larvae reared on natural pollen from conspecific (natural *O. lignaria* pollen) and heterospecific (natural *O. ribifloris* pollen) sources (Figure 2b; Table S3).

Findings from the Cox regression analysis revealed that for *O. ribifloris*, the Omnibus test indicated a significant improvement in fit for the current model relative to the null (χ^2^_(2)_ = 22.87, *p* < .001). There was a significant positive regression coefficient for the hazard rate (B = 3.39, SE = 1.06, *p* = .002, Exp(B) = 26.91) for pollen‐borne microbes indicating that for *O. ribifloris*, lack of microbes represented a significant increase in hazard for death. There was a positive regression coefficient for the hazard rate (B = 0.30, SE = 0.48, *p* = .55, Exp(B) = 1.35) for source of pollen implying that heterospecific‐sourced pollen represented a greater hazard for death, although this relationship was not statistically significant. Similar findings were observed for *O. lignaria*, wherein the Omnibus test indicated a significant improvement in fit for the current model relative to the null (χ^2^_(2)_ = 31.20, *p* < .001). There was a significant positive regression coefficient for the hazard rate (B = 3.03, SE = 0.68, *p* < .001, Exp(B) = 20.63) for pollen‐borne microbes indicating that for *O. lignaria*, lack of microbes represented a significant increase in hazard for death. There was a positive regression coefficient for the hazard rate (B = 0.90, SE = 0.48, *p* = .06, Exp(B) = 2.47) for source of pollen implying that heterospecific‐sourced pollen represented a greater hazard for death, although this relationship was just above the level of statistical significance.

**FIGURE 3:**
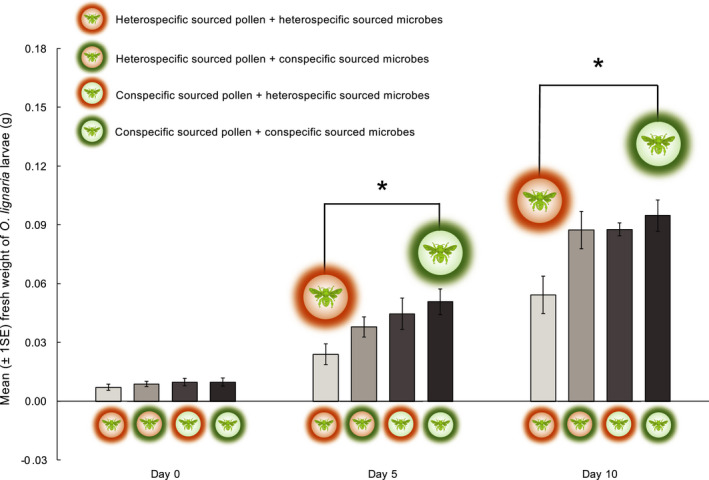
Mean fresh weights of *Osmia lignaria* larvae (±1 SE) measured over three time points across four diet treatments. Inset symbols above bars represent pairwise comparisons of larval weights across diet treatments within a given time increment (*p < .05)

*Study 2*: Results from the repeated measures ANOVA (Greenhouse–Geisser correction ε = 0.63, χ^2^_(2)_ = 25.72, *p* < .001) indicated that the interaction between time and treatment had a significant effect on larval biomass (*F*_3.78, 37.78_ = 3.61, *p* = .015). The main effects of time (*F*_1.23, 37.78_ = 253.38, *p* < .001) and diet treatment (*F*_3,30_ = 3.45, *p* = .03) were also significant. Pairwise post hoc tests indicated that while the initial larval biomass was comparable across treatments, for all subsequent time points, larvae reared on sterilized conspecific‐sourced pollen with conspecific‐sourced microbes (sterilized *O. lignaria* pollen inoculated with *O. lignaria* microbes) had significantly higher biomass than those reared on sterilized heterospecific‐sourced pollen with heterospecific‐sourced microbes (sterilized *O. cornifrons* pollen inoculated with *O. cornifrons* microbes) (Figure 3, Table S4).

**FIGURE 4:**
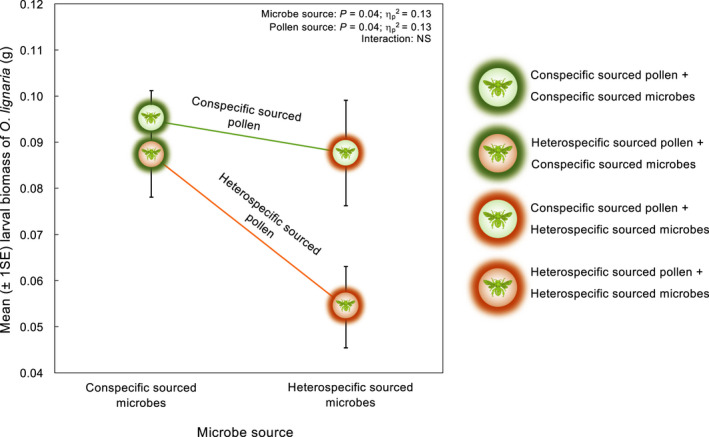
Interaction plot showing the impact of pollen source and microbe source on the biomass of surviving *Osmia lignaria* larvae by day 10

A two‐way ANOVA revealed a significant main effect of source of pollen, with larvae consuming conspecific‐sourced pollen and heterospecific‐sourced pollen weighing 0.09 ± 0.04 g (*N* = 16) and 0.07 ± 0.04 g (*N* = 18), respectively. For larvae reared on conspecific‐sourced pollen (sterilized *O. lignaria* pollen), there was no difference in biomass based on the source of microbes (pairwise contrast *F*_1,30_ = 0.26, *p* = .61). However, for heterospecific‐sourced pollen (sterilized *O. cornifrons* pollen), larvae weighed significantly more in the presence of conspecific‐sourced microbes (*O. lignaria* microbes) than heterospecific‐sourced microbes (*O. cornifrons* microbes) (pairwise contrast *F*_1,30_ = 6.45, *p* = .02). The source of microbes also had a significant main effect, with larvae reared in the presence of conspecific‐sourced microbes and heterospecific‐sourced microbes weighing 0.09 ± 0.04 g (*N* = 16) and 0.07 ± 0.04 g (*N* = 18), respectively. When conspecific‐sourced microbes (*O. lignaria* microbes) were present within their diets, larval biomass did not vary based on pollen source (pairwise contrast *F*_1,30_ = 0.29, *p* = .56). However, when heterospecific‐sourced microbes (*O. cornifrons* microbes) were present within their diets, larval biomass was significantly higher among those reared on conspecific‐sourced pollen (sterilized *O. lignaria* pollen) than heterospecific‐sourced pollen (sterilized *O. cornifrons* pollen) (pairwise contrast *F*_1,30_ = 6.56, *p* = .02) (Figure 4). Estimates of effect size based on the Hedges’ g (Lakens, 2013) indicated that when fed conspecific‐sourced pollen (sterilized *O. lignaria* pollen), the source of microbes had a trivial impact on larval biomass (*g_conspecific_pollen_* = 0.09). In contrast, when fed heterospecific‐sourced pollen (sterilized *O. cornifrons* pollen), larvae reared in the presence of heterospecific‐sourced microbes (*O. cornifrons* microbes) weighed significantly less than those reared in the presence of conspecific‐sourced microbes (*O. lignaria* microbes) (*g_heterospecific_pollen_* = 1.09). Furthermore, inoculation of larval diets with conspecific‐sourced microbes (*O. lignaria* microbes) had trivial impact on larval performance across pollen sources (*g_conspecific_microbes_* = 0.31). Conversely, inoculation of larval diets with heterospecific‐sourced microbes (*O. cornifrons* microbes) had a large effect on impact on larval performance (*g_heterospecific_microbes_* = 1.09), where larvae fed heterospecific‐sourced pollen (sterilized *O. cornifrons* pollen) weighed significantly less than those fed conspecific‐sourced pollen (sterilized *O. lignaria* pollen).

## 4 DISCUSSION

Two studies were conducted to investigate the importance of microbial exosymbionts for solitary bee development. The first study examined whether the presence or absence of microbial exosymbionts was as important for polyleges as oligoleges. The expectation from Study 1 was that if microbial exosymbionts were truly critical for the development of solitary bee larvae, this effect should be consistent across taxa and across foraging strategies. We also predicted that the magnitude of the effect size of exosymbionts would be stronger among oligolectic larvae. In the second study, the importance of microbial sourcing was examined concurrently with that of pollen sourcing to ascertain the relative importance of each of these factors for brood success. In contrast to Study 1 (which investigated the effect of microbe presence/absence), all diet treatments in Study 2 contained microbes inoculated within larval pollen provisions. However, the source of the microbes was manipulated, allowing us to compare the effects of having conspecific‐sourced microbes (i.e., microbes associated with pollen provision allocated by the mother bee of the same species) versus heterospecific‐sourced microbes (i.e., microbes associated with pollen provision allocated by the mother bee of a different species) within larval diets.

Results from our first study reveal that the microbes associated with larval pollen provisions were critical for the development of both the oligolege (*O. ribifloris*) and polylege *(O. lignaria)*. This corroborates and extends the findings of previously published research, which documented the importance of pollen‐borne microbes for the development of oligolectic larvae (Dharampal et al., 2019; Dharampal, Hetherington, et al., 2020), indicating that the same might be true for polyleges as well. Whether allocated by a conspecific or heterospecific foraging female, provisions that were accompanied by their natural microbiota resulted in high‐performing larvae. Both *O. ribifloris* and *O. lignaria* larvae took approximately the same time to complete larval development and reached comparable prepupal biomass when reared on their own pollen or that of the other species, as long as microbes were present. In contrast, the lack of microbes in pollen provisions led to severe brood failure among both species. When reared on pollen that was devoid of microbes, larvae from both species suffered lowered fitness, regardless of whether they were fed pollen sourced from an adult forager of the same species or from the other. This suggests that for both oligolectic and polylectic solitary bees, microbes present within the pollen provision were likely critical for larval survival, regardless of whether the pollen was provisioned by a conspecific or heterospecific forager. Previous work suggests that this association between pollen‐associated microbes and larval health may be attributed to the nutritional symbioses between the two. For instance, trophic reconstruction studies using biomarker‐based assays have previously revealed that microbial exosymbionts represent nutritional mutualists and direct prey items that facilitate nutrient transfer from pollen provision to larval bees, dramatically improving brood outcome (Dharampal et al., 2019; Dharampal, Hetherington, et al., 2020; Steffan et al., 2019). These studies have empirically quantified microbially derived proteins and lipids within bee biomass, reporting that pollen‐associated microbes form a dominant source of nutrition for developing larvae. Our study corroborates and extends these findings to include a polylectic species, suggesting that larval reliance on their exosymbionts may be more ubiquitous among solitary bees, regardless of their foraging strategy.

The importance of microbes was also reflected in the survival outcome for both the oligolege and polylege, with the presence of microbes profoundly improving survivorship components. Larval survivorship varied significantly based on treatment type; while 90% of the larvae reared in the presence of pollen‐associated microbes reached the prepupal stage, survivorship declined dramatically to 10% among those reared on microbe‐deficient diets. Whether reared on conspecific‐ or heterospecific‐sourced pollen, larvae of both species suffered significantly higher mortality when microbes were lacking from their diet. In fact, the worst survivorship outcome for both species was noted among larvae reared on heterospecific‐sourced pollen without microbes (i.e., *O. lignaria* on sterilized *O. ribifloris* pollen, and vice versa), where none of the larvae survived to the prepupal stage. In contrast, survivorship improved significantly among larvae reared on microbe‐rich pollen, and was comparable for both heterospecific and conspecific pollen sources. This pattern was consistent for the oligolege as well as the polylege, suggesting that the availability of microbes within larval provisions may have been a stronger predictor of brood survival than pollen source for both types of foragers (Figure 2). Furthermore, hazard analysis based on larval time to death revealed that, unlike pollen source, the absence of microbes represented a severe and significant risk for larval survival. However, the magnitude of the hazard varied across foraging strategies; the risk of death among oligoleges when reared on sterilized diets increased 27 times compared to 20 times for the polylege. This indicated that oligoleges are more susceptible to the absence of pollen‐borne microbiota, presumably due to increased reliance on nutritional exosymbionts associated with their low‐quality conspecific pollen, and this was consistent with earlier findings (Dharampal, Hetherington, et al., 2020). Interestingly, survivorship outcome for both species was unaffected by the source of pollen and neither species showed any significant increase in the risk of death when fed pollen that was heterospecific‐sourced instead of conspecific‐sourced. Taken together, these findings strongly imply that the absence of microbes may have a profound adverse impact on larval performance, and that this effect persists across foraging mode and pollen source.

For the second study, we examined the importance of the source of microbes along with that of forage pollen for the development of *O. lignaria* larvae. Given that microbes were always present in the pollen provisions, and that the microbial and pollen compositions were sourced from either conspecific or heterospecific bees, we could examine the main and interactive effects of microbial and pollen sourcing. Ostensibly, pollen provisions allocated by conspecific foragers would comprise the ‘right kind’ of pollen composition, accompanied with the ‘right kind’ of naturally occurring microbiota for their progeny. In contrast, the pollen composition provided by heterospecific foragers could be considered the ‘wrong kind’ of pollen, and the microbes embedded therein, also of the ‘wrong kind’. Thus, any given *O. lignaria* larva in the second study was fed either pollen of the right kind (sterilized *O. lignaria* pollen) or of the wrong kind (sterilized *O. cornifrons* pollen) that was colonized by either microbes of the right kind (microbes sourced from *O. lignaria* pollen) or the wrong kind (microbes sourced from *O. cornifrons* pollen).

Growth rate analysis indicated that larval development among polyleges was strongly impacted by the source of pollen and microbes afforded in their diet. Although all larvae had comparable weights at the start of the study, larval biomass began to show significant differences as early as day 5. Over the course of 10 days, the disparity between larvae that received the right microbes and right pollen (i.e., conspecific foraging) versus larvae that received the wrong microbes and wrong pollen (i.e., heterospecific foraging) increased markedly (Figure 3). This implied that for developing bees, the symbioses with their microbial partners were most beneficial when provisions were sourced from a conspecific female paired with the natural conspecific microbiota. Analyses of microbial and pollen sourcing indicated that both the source of pollen and that of the microbes were significant drivers of bee fitness (Figure 4). Furthermore, the impact of each was almost identical, suggesting that the microbial community in a pollen provision was just as important for bee development as the pollen itself.

In manipulating the source of microbes within larval diet, we observed that microbial sourcing had a large impact on larval fitness when pollen was sourced from a heterospecific forager, but not from that of a conspecific. While larvae reared on heterospecific‐sourced pollen along with the innate heterospecific‐sourced microbes suffered a marked decline in fitness components, those consuming heterospecific‐sourced pollen inoculated with conspecific‐sourced microbes showed a significant increase in biomass. This implied that for larvae consuming the ‘wrong’ pollen, replacing the ‘wrong’ heterospecific‐sourced microbes with the ‘right’ conspecific‐sourced ones may have had a strong positive effect on larval health. One explanation for these findings based on previously published studies is that conspecific‐sourced microbes likely perform important nutritive functions, such as pollen fermentation and nutrient transfer (Steffan & Dharampal, 2019; Voulgari‐Kokota, McFrederick, et al., 2019), enhancing the accessibility of nutrients within heterospecific‐sourced pollen. Interestingly, the compensatory effects of conspecific‐sourced microbes did not extend to conspecific‐sourced pollen. Biomass of larvae reared on conspecific‐sourced pollen remained comparable among treatments regardless of whether microbes were sourced from a conspecific or heterospecific. The minimal impact of microbial sourcing indicated that larvae may be physiologically better adapted to exploiting the conspecific pollen substrate and are thus, less sensitive to the taxonomic specificity of the microbiota present therein. Taken together, these results indicate that the emergent beneficial effect of the partnership between bees and their exosymbionts is greatest when both the right kind of pollen and and right kind of microbes are available, and declines progressively as one or both components are eliminated.

Given the minimal scope of vertical transmission from nest mates, the microbiome within solitary bee pollen provisions is largely driven by the local environment (McFrederick et al., 2012; Voulgari‐Kokota et al., 2018) and tends to fluctuate based on bee species (Keller et al., 2013; Lozo et al., 2015), floral transmission routes (McFrederick et al., 2016), foraging tendencies (Voulgari‐Kokota, Ankenbrand, et al., 2019), and pollen usage across habitats (McFrederick & Rehan, 2019). Another important source of variation is the diet breadth of individual species based on their pollen specialization strategy (Keller et al., 2020). For instance, as an Ericaceae specialist with a narrow host plant range, *O. ribifloris* is likely to acquire a distinct community of microbes compared to the generalist, *O. lignaria*, that forages on a broader diversity of orchard trees (Rothman et al., 2020). Such differences in host plant preferences likely expose the two species to specific microbial taxa which possess specialized functional adaptations to the respective pollen types (e.g., detoxification of secondary metabolites). Additionally, the unique microenvironments of oligolectic and polylectic provisions can preferentially filter microbes based on the nutritional chemistry of pollen and nectar, thereby shaping the community composition within the provisions (Keller et al., 2020). Indeed, recent findings indicate minimal overlap between the microbial communities associated with the pollen provisions of *O. ribifloris* and *O. lignaria*, the differences likely being driven by a combination of factors such as diet breath, local environment, and nest‐building materials (Rothman et al., 2019, 2020). Collectively, these studies suggest that both bee species host a taxonomically unique community of well‐adapted microbes within their pollen provisions. Although comparing the microbial community associated with both bee species fell beyond the scope of this study, our findings reveal that notwithstanding their taxonomic specificity, the conspecific exosymbionts of both the polylege and oligolege are significant determinants of bee fitness outcome.

While our study offers compelling evidence supporting the function of exosymbiotic microbes in shaping bee fitness, a possible alternative explanation of our results is that the sterilization process may have compromised the nutritional value of pollen, confounding our findings. However, past studies did not find any significant difference between the nutrient profile of sterilized versus unsterilized pollen (Dharampal et al., 2019; Dharampal, Hetherington, et al., 2020). Another study using laboratory‐reared bumblebees showed significant colony growth when fed sterilized pollen that was recolonized by non‐pathogenic microbes (Steffan et al., 2017). This suggested that the sterilization technique itself, did not produce any measurable adverse effect on pollen nutrient composition or the fitness outcome of bees that consumed pollen sterilized in this manner. Thus, based on prior research and direct quantification, it is unlikely that the sterilization of pollen resulted in a marked decline in diet quality, leading to the trends reported here. Another potential limitation of our study is that we did not investigate the extent to which larval digestive physiology may have influenced our results. The ability of larval bees to digest different pollen types may depend on their metabolic capabilities. However, if larval nutrition was solely driven by their intrinsic metabolic capacity to digest pollen, it would not explain the dramatic mortality among larvae that were offered ample amounts of conspecific‐sourced pollen, but not the pollen‐associated microbiota. Another facypossible limitation of our study is that we did not ascertain the extent of microbial recolonization in Study 2. Our data reveal that larvae reared on sterilized diets, which were inoculated with microbes, showed high survivorship compared to those on sterilized diets, which were not inoculated. Thus, the difference in bee survival was likely mediated by the symbiotic pollen‐associated microbes and this was strongly indicative of successful microbial recolonization of the sterilized pollen. We also acknowledge that our study investigated a single representative species of both foraging strategies among solitary bees. Moreover, since our study design required a large number of bees for adequate replication, we elected to use the significantly more abundant male progeny. Given the differences in life history traits, it would be interesting to investigate whether our findings would vary based on gender. Further studies using males and females from additional representative oligolectic and polylectic species will be needed to establish the potential functions of conspecific microbiota for the bee species hosting them.

The symbioses between bees and microbes represent one of the major paradigms in insect–microbe interactions. Yet, the relationship between solitary bees and their exosymbionts has remained poorly resolved. Our study contributes to this existing knowledge gap by demonstrating that the identity of microbial symbionts within pollen provisions is just as critical for larval development as the pollen source itself. Findings presented here indicate that the appropriate pairing of conspecific‐sourced microbes with conspecific‐sourced pollen yields the greatest benefit for developing solitary bees than either component by itself—a phenomenon that appears to be consistent across oligoleges and polyleges alike. This represents strong evidence that there is some degree of specificity between a given bee species, its particular pollen diet, and the natural microbiota therein. Thus, if there are disruptions to this innate coupling via external stressors, it could cause severe declines in bee fitness. For instance, exposure to fungicides during foraging trips can contaminate larval pollen provisions, leading to elevated concentrations of fungicide residues within nest‐stored pollen (Artz & Pitts‐Singer, 2015; Sgolastra et al., 2017, 2018). This can cause detrimental alterations to the symbiotic microbial community by removing beneficial taxa and/or by increasing susceptibility to opportunistic pathogens (Steffan et al., 2017). Given that our study identifies pollen‐associated microbiota as being just as important as the identity of the forage pollen itself, conserving the partnership between bees and their exosymbionts will be critical for maintaining healthy bee populations.

## CONFLICT OF INTEREST

The authors have no conflict of interest to declare.

## AUTHOR CONTRIBUTIONS

**Prarthana S. Dharampal:** Conceptualization (equal); Formal analysis (equal); Investigation (equal); Methodology (equal); Visualization (equal); Writing – original draft (equal). **Bryan N. Danforth:** Resources (equal); Validation (supporting); Writing – review & editing (equal). **Shawn A. Steffan:** Conceptualization (equal); Formal analysis (supporting); Funding acquisition (lead); Investigation (equal); Methodology (equal); Project administration (lead); Resources (lead); Supervision (lead); Validation (equal); Writing – original draft (equal).

## Supporting information

Supplementary Material

## Data Availability

Additional data and electronic supplementary material can be viewed at https://doi.org/10.5061/dryad.3ffbg79kx.
