# Supplementary material for: Exosymbiotic microbes within fermented pollen provisions are as important for the development of solitary bees as the pollen itself
Source: Ecol Evol. 2022 Apr 6;12(4):e8788. doi: 10.1002/ece3.8788 (PMC8986510; doi:10.1002/ece3.8788)
Supplement: Supplementary file 1 — Supplementary Material [file ECE3-12-e8788-s001.docx]

Supplemental Information

Exosymbiotic microbes within fermented pollen provisions are as important for the development of solitary bees as the pollen itself

Prarthana S. Dharampal^1*^, Bryan N. Danforth^2^ and Shawn A. Steffan^1,3^

^1^Department of Entomology, University of Wisconsin, 1630 Linden Dr, Madison, WI 53706, USA

^2^Department of Entomology, Cornell University, 2126 Comstock Hall, Ithaca, NY 14853, USA

^3^USDA-ARS, Vegetable Crops Research Unit, 1575 Linden Dr, Madison, WI 53706, USA

*Corresponding author:

1630 Linden Drive, Madison, Wisconsin 53706

Ph no: 608-890-128; email: [pghosh6@wisc.edu](mailto:pghosh6@wisc.edu)

| Table S1. Description of diet treatments for Study 1 | | | | | | | |  |  |
| --- | --- | --- | --- | --- | --- | --- | --- | --- | --- |
|  |  |  |  |  |  |  |  |  |  |
|  |  |  |  |  |  |  |  |  |  |
| Larva | Pollen type | Pollen-borne microbes | Pollen specialization | FW of natural *O. ribifloris* pollen (g) | DW of sterile *O. ribifloris* pollen (g) | FW of natural *O. lignaria* pollen (g) | DW of sterile *O. lignaria* pollen (g) | Vol. of sterile water added (µl) |  |
|  |  |  |  |  |  |  |  |  |  |
| *O. ribifloris* | Conspecific-sourced | Absent | Oligolecty | 0 | 0.28 | 0 | 0 | 70 |  |
|  | Heterospecific-sourced | Absent | Oligolecty | 0 | 0 | 0 | 0.28 | 70 |  |
|  | Conspecific-sourced | Present | Oligolecty | 0.35 | 0 | 0 | 0 | 0 |  |
|  | Heterospecific-sourced | Present | Oligolecty | 0 | 0 | 0.35 | 0 | 0 |  |
| *O. lignaria* | Conspecific-sourced | Absent | Polylecty | 0 | 0 | 0 | 0.32 | 50 |  |
|  | Heterospecific-sourced | Absent | Polylecty | 0 | 0.32 | 0 | 0 | 50 |  |
|  | Conspecific-sourced | Present | Polylecty | 0 | 0 | 0.37 | 0 | 0 |  |
|  | Heterospecific-sourced | Present | Polylecty | 0.37 | 0 | 0 | 0 | 0 |  |

| Table S2. Description of diet treatments for Study 2 | | | | | | |  |  |  |  |
| --- | --- | --- | --- | --- | --- | --- | --- | --- | --- | --- |
|  |  |  |  |  |  |  |  |  |  |  |
| Larva | Pollen source | Source of pollen-borne microbes | Dry weight of sterile conspecific-sourced pollen (g) | Fresh weight of natural conspecific-sourced pollen (g) | Dry weight of sterile heterospecific- sourced pollen (g) | Fresh weight of natural heterospecific-sourced pollen (g) | Weight of sterile water added (g) | Volume of sterile water added (ul) | Final fresh weight of provision |  |
|  |  |  |  |  |  |  |  |  |  |  |
| *O. lignaria* | Conspecific-sourced | Conspecific-sourced | 0.256 | 0.074 | 0 | 0 | 0.04 | 40 | 0.37 |  |
|  | Heterospecific-sourced | Conspecific-sourced | 0 | 0.074 | 0.256 | 0 | 0.04 | 40 | 0.37 |  |
|  | Conspecific-sourced | Heterospecific-sourced | 0.256 | 0 | 0 | 0.074 | 0.04 | 40 | 0.37 |  |
|  | Heterospecific-sourced | Heterospecific-sourced | 0 | 0 | 0.256 | 0.074 | 0.04 | 40 | 0.37 |  |
|  |  |  |  |  |  |  |  |  |  |  |

| Table S3. Post hoc comparisons of survival probability of *O. ribifloris* and *O. lignaria* across diet treatment from Study 1 | | | | | | | |  |  |
| --- | --- | --- | --- | --- | --- | --- | --- | --- | --- |
|  |  |  |  |  |  |  |  |  |  |
|  |  |  |  |  |  |  |  |  |  |
|  |  | *O. ribifloris* on sterile conspecific-sourced pollen | | *O. ribifloris* on sterile heterospecific-sourced pollen | | *O. ribifloris* on natural conspecific-sourced pollen | | *O. ribifloris* on natural heterospecific-sourced pollen | |
| Test | Treatment | Chi-Square | Sig. | Chi-Square | Sig. | Chi-Square | Sig. | Chi-Square | Sig. |
| Log rank (Mantel-Cox) | *O. ribifloris* on sterile conspecific-sourced pollen |  |  | 4.750 | 0.029 | 9.726 | 0.002 | 13.520 | 0.000 |
|  | *O. ribifloris* on sterile heterospecific-sourced pollen | 4.750 | 0.029 |  |  | 15.545 | 0.000 | 19.000 | 0.000 |
|  | *O. ribifloris* on natural conspecific-sourced pollen | 9.726 | 0.002 | 15.545 | 0.000 |  |  | 1.000 | 0.317 |
|  | *O. ribifloris* on heterospecific-sourced pollen | 13.520 | 0.000 | 19.000 | 0.000 | 1.000 | 0.317 |  |  |
|  |  |  |  |  |  |  |  |  |  |
| Test | Treatment | Wilcoxon Statistic | Sig. | Wilcoxon Statistic | Sig. | Wilcoxon Statistic | Sig. | Wilcoxon Statistic | Sig. |
| Wilcoxon (Gehan) Statistic | *O. ribifloris* on sterile conspecific-sourced pollen |  |  | 4.684 | 0.030 | 8.335 | 0.004 | 12.052 | 0.001 |
|  | *O. ribifloris* on sterile heterospecific-sourced pollen | 4.684 | 0.030 |  |  | 15.545 | 0.000 | 19.000 | 0.000 |
|  | *O. ribifloris* on natural conspecific-sourced pollen | 8.335 | 0.004 | 15.545 | 0.000 |  |  | 1.000 | 0.317 |
|  | *O. ribifloris* on natural heterospecific-sourced pollen | 12.052 | 0.001 | 19.000 | 0.000 | 1.000 | 0.317 |  |  |
|  |  |  |  |  |  |  |  |  |  |
|  |  | *O. lignaria* on sterile conspecific-sourced pollen | | *O. lignaria* on sterile heterospecific-sourced pollen | | *O. lignaria* on natural conspecific-sourced pollen | | *O. lignaria* on natural heterospecific-sourced pollen | |
| Test | Treatment | Chi-Square | Sig. | Chi-Square | Sig. | Chi-Square | Sig. | Chi-Square | Sig. |
| Log rank (Mantel-Cox) | *O. lignaria* on sterile conspecific-sourced pollen |  |  | 8.695 | 0.003 | 14.864 | 0.000 | 12.930 | 0.000 |
|  | *O. lignaria* on sterile heterospecific-sourced pollen | 8.695 | 0.003 |  |  | 17.079 | 0.000 | 17.165 | 0.000 |
|  | *O. lignaria* on natural conspecific-sourced pollen | 14.864 | 0.000 | 17.079 | 0.000 |  |  | 0.299 | 0.584 |
|  | *O. lignaria* on natural heterospecific-sourced pollen | 12.930 | 0.000 | 17.165 | 0.000 | 0.299 | 0.584 |  |  |
|  |  |  |  |  |  |  |  |  |  |
| Test | Treatment | Wilcoxon Statistic | Sig. | Wilcoxon Statistic | Sig. | Wilcoxon Statistic | Sig. | Wilcoxon Statistic | Sig. |
| Wilcoxon (Gehan) Statistic | *O. lignaria* on sterile conspecific-sourced pollen |  |  | 8.479 | 0.004 | 10.700 | 0.001 | 10.873 | 0.001 |
|  | *O. lignaria* on sterile heterospecific-sourced pollen | 8.479 | 0.004 |  |  | 14.402 | 0.000 | 15.551 | 0.000 |
|  | *O. lignaria* on natural conspecific-sourced pollen | 10.700 | 0.001 | 14.402 | 0.000 |  |  | 0.237 | 0.627 |
|  | *O. lignaria* on natural heterospecific-sourced pollen | 10.873 | 0.001 | 15.551 | 0.000 | 0.237 | 0.627 |  |  |

| Table S4. Pairwise comparisons of repeated measures analysis of larval biomass of *O. lignaria* across diet treatments from Study 2 | | | | | | | |
| --- | --- | --- | --- | --- | --- | --- | --- |
|  |  |  |  |  |  | 95% Confidence Interval for Difference^b^ | |
| Time | (I) TREATMENT | (J) TREATMENT | Mean Difference (I-J) | Std. Error | Sig.^b^ | Lower Bound | Upper Bound |
| **Day 0** | Conspecific-sourced pollen + Heterospecific-sourced microbes | Heterospecific-sourced pollen + Heterospecific-sourced microbes | 0.003 | 0.002 | 1.000 | -0.004 | 0.010 |
|  |  | Conspecific-sourced pollen + Conspecific-sourced microbes | -0.002 | 0.003 | 1.000 | -0.010 | 0.005 |
|  |  | Heterospecific-sourced pollen + Conspecific-sourced microbes | 0.001 | 0.002 | 1.000 | -0.006 | 0.008 |
|  | Heterospecific-sourced pollen + Heterospecific-sourced microbes | Conspecific-sourced pollen + Heterospecific-sourced microbes | -0.003 | 0.002 | 1.000 | -0.010 | 0.004 |
|  |  | Conspecific-sourced pollen + Conspecific-sourced microbes | -0.005 | 0.003 | 0.319 | -0.012 | 0.002 |
|  |  | Heterospecific-sourced pollen + Conspecific-sourced microbes | -0.002 | 0.002 | 1.000 | -0.008 | 0.005 |
|  | Conspecific-sourced pollen + Conspecific-sourced microbes | Conspecific-sourced pollen + Heterospecific-sourced microbes | 0.002 | 0.003 | 1.000 | -0.005 | 0.010 |
|  |  | Heterospecific-sourced pollen + Heterospecific-sourced microbes | 0.005 | 0.003 | 0.319 | -0.002 | 0.012 |
|  |  | Heterospecific-sourced pollen + Conspecific-sourced microbes | 0.004 | 0.003 | 1.000 | -0.004 | 0.011 |
|  | Heterospecific-sourced pollen + Conspecific-sourced microbes | Conspecific-sourced pollen + Heterospecific-sourced microbes | -0.001 | 0.002 | 1.000 | -0.008 | 0.006 |
|  |  | Heterospecific-sourced pollen + Heterospecific-sourced microbes | 0.002 | 0.002 | 1.000 | -0.005 | 0.008 |
|  |  | Conspecific-sourced pollen + Conspecific-sourced microbes | -0.004 | 0.003 | 1.000 | -0.011 | 0.004 |
| **Day 5** | Conspecific-sourced pollen + Heterospecific-sourced microbes | Heterospecific-sourced pollen + Heterospecific-sourced microbes | 0.021 | 0.009 | 0.151 | -0.004 | 0.045 |
|  |  | Conspecific-sourced pollen + Conspecific-sourced microbes | -0.006 | 0.009 | 1.000 | -0.033 | 0.020 |
|  |  | Heterospecific-sourced pollen + Conspecific-sourced microbes | 0.007 | 0.009 | 1.000 | -0.018 | 0.031 |
|  | Heterospecific-sourced pollen + Heterospecific-sourced microbes | Conspecific-sourced pollen + Heterospecific-sourced microbes | -0.021 | 0.009 | 0.151 | -0.045 | 0.004 |
|  |  | Conspecific-sourced pollen + Conspecific-sourced microbes | -.027^*^ | 0.009 | **0.046** | -0.053 | 0.000 |
|  |  | Heterospecific-sourced pollen + Conspecific-sourced microbes | -0.014 | 0.009 | 0.737 | -0.039 | 0.011 |
|  | Conspecific-sourced pollen + Conspecific-sourced microbes | Conspecific-sourced pollen + Heterospecific-sourced microbes | 0.006 | 0.009 | 1.000 | -0.020 | 0.033 |
|  |  | Heterospecific-sourced pollen + Heterospecific-sourced microbes | .027^*^ | 0.009 | **0.046** | 0.000 | 0.053 |
|  |  | Heterospecific-sourced pollen + Conspecific-sourced microbes | 0.013 | 0.009 | 1.000 | -0.014 | 0.039 |
|  | Heterospecific-sourced pollen + Conspecific-sourced microbes | Conspecific-sourced pollen + Heterospecific-sourced microbes | -0.007 | 0.009 | 1.000 | -0.031 | 0.018 |
|  |  | Heterospecific-sourced pollen + Heterospecific-sourced microbes | 0.014 | 0.009 | 0.737 | -0.011 | 0.039 |
|  |  | Conspecific-sourced pollen + Conspecific-sourced microbes | -0.013 | 0.009 | 1.000 | -0.039 | 0.014 |
| **Day 10** | Conspecific-sourced pollen + Heterospecific-sourced microbes | Heterospecific-sourced pollen + Heterospecific-sourced microbes | 0.033 | 0.013 | 0.093 | -0.003 | 0.070 |
|  |  | Conspecific-sourced pollen + Conspecific-sourced microbes | -0.007 | 0.014 | 1.000 | -0.046 | 0.032 |
|  |  | Heterospecific-sourced pollen + Conspecific-sourced microbes | 0.000 | 0.013 | 1.000 | -0.036 | 0.037 |
|  | Heterospecific-sourced pollen + Heterospecific-sourced microbes | Conspecific-sourced pollen + Heterospecific-sourced microbes | -0.033 | 0.013 | 0.093 | -0.070 | 0.003 |
|  |  | Conspecific-sourced pollen + Conspecific-sourced microbes | -.040^*^ | 0.014 | **0.041** | -0.080 | -0.001 |
|  |  | Heterospecific-sourced pollen + Conspecific-sourced microbes | -0.033 | 0.013 | 0.099 | -0.070 | 0.004 |
|  | Conspecific-sourced pollen + Conspecific-sourced microbes | Conspecific-sourced pollen + Heterospecific-sourced microbes | 0.007 | 0.014 | 1.000 | -0.032 | 0.046 |
|  |  | Heterospecific-sourced pollen + Heterospecific-sourced microbes | .040^*^ | 0.014 | **0.041** | 0.001 | 0.080 |
|  |  | Heterospecific-sourced pollen + Conspecific-sourced microbes | 0.007 | 0.014 | 1.000 | -0.032 | 0.047 |
|  | Heterospecific-sourced pollen + Conspecific-sourced microbes | Conspecific-sourced pollen + Heterospecific-sourced microbes | 0.000 | 0.013 | 1.000 | -0.037 | 0.036 |
|  |  | Heterospecific-sourced pollen + Heterospecific-sourced microbes | 0.033 | 0.013 | 0.099 | -0.004 | 0.070 |
|  |  | Conspecific-sourced pollen + Conspecific-sourced microbes | -0.007 | 0.014 | 1.000 | -0.047 | 0.032 |
| Based on estimated marginal means | |  |  |  |  |  | |
| *. The mean difference is significant at the .05 level. | | |  |  |  |  | |
| b. Adjustment for multiple comparisons: Bonferroni. | | | | | | | |
